# Behavioral Intention to Receive a COVID-19 Vaccination Among Chinese Factory Workers: Cross-sectional Online Survey

**DOI:** 10.2196/24673

**Published:** 2021-03-09

**Authors:** Ke Chun Zhang, Yuan Fang, He Cao, Hongbiao Chen, Tian Hu, Yaqi Chen, Xiaofeng Zhou, Zixin Wang

**Affiliations:** 1 Longhua District Center for Disease Control and Prevention Shenzhen China; 2 Department of Early Childhood Education The Education University of Hong Kong Hong Kong China; 3 JC School of Public Health and Primary Care The Chinese University of Hong Kong Hong Kong China

**Keywords:** COVID-19, vaccination, behavioral intention, perception, social media influence, personal preventive behaviors, factory workers, China, social media, vaccine, behavior, intention, risk

## Abstract

**Background:**

COVID-19 vaccines will become available in China soon. Understanding communities’ responses to the forthcoming COVID-19 vaccines is important. We applied the theory of planned behavior as the theoretical framework.

**Objective:**

This study investigates the prevalence of and factors associated with behavioral intention to receive self-financed or free COVID-19 vaccinations among Chinese factory workers who resumed work during the pandemic. We examined the effects of factors including sociodemographics, perceptions related to COVID-19 vaccination, exposure to information about COVID-19 vaccination through social media, and COVID-19 preventive measures implemented by individuals and factories.

**Methods:**

Participants were full-time employees 18 years or older who worked in factories in Shenzhen. Factory workers in Shenzhen are required to receive a physical examination annually. Eligible workers attending six physical examination sites were invited to complete a survey on September 1-7, 2020. Out of 2653 eligible factory workers, 2053 (77.4%) completed the online survey. Multivariate two-level logistic regression models and ordinal logistic regression models were fitted.

**Results:**

The prevalence of behavioral intention to receive a COVID-19 vaccination was 66.6% (n=1368, conditional on 80% vaccine efficacy and market rate) and 80.6% (n=1655, conditional on 80% vaccine efficacy and free vaccines). After adjusting for significant background characteristics, positive attitudes toward COVID-19 vaccination (adjusted odds ratio [AOR] 1.20, 95% CI 1.15-1.25 and AOR 1.24, 95% CI 1.19-1.30), perceived support from significant others for getting a COVID-19 vaccination (AOR 1.43, 95% CI 1.32-1.55 and AOR 1.37, 95% CI 1.25-1.50), and perceived behavioral control to get a COVID-19 vaccination (AOR 1.51, 95% CI 1.32-1.73 and AOR 1.28, 95% CI 1.09-1.51) were positively associated with both dependent variables (conditional on 80% vaccine efficacy and market rate or free vaccines, respectively). Regarding social media influence, higher frequency of exposure to positive information related to COVID-19 vaccination was associated with a higher intention to receive a COVID-19 vaccination at market rate (AOR 1.53, 95% CI 1.39-1.70) or a free vaccination (AOR 1.52, 95% CI 1.35-1.71). Higher self-reported compliance with wearing a face mask in the workplace (AOR 1.27, 95% CI 1.02-1.58 and AOR 1.67, 95% CI 1.24-2.27) and other public spaces (AOR 1.80, 95% CI 1.42-2.29 and AOR 1.34, 95% CI 1.01-1.77), hand hygiene (AOR 1.21, 95% CI 1.00-1.47 and AOR 1.52, 95% CI 1.19-1.93), and avoiding social gatherings (AOR 1.22, 95% CI 1.01-1.47 and AOR 1.55, 95% CI 1.23-1.95) and crowded places (AOR 1.24, 95% CI 1.02-1.51 and AOR 1.73, 95% CI 1.37-2.18) were also positively associated with both dependent variables. The number of COVID-19 preventive measures implemented by the factory was positively associated with the intention to receive a COVID-19 vaccination under both scenarios (AOR 1.08, 95% CI 1.04-1.12 and AOR 1.06, 95% CI 1.01-1.11).

**Conclusions:**

Factory workers in China reported a high behavioral intention to receive a COVID-19 vaccination. The theory of planned behavior is a useful framework to guide the development of future campaigns promoting COVID-19 vaccination.

## Introduction

The COVID-19 pandemic remains out of control worldwide. As of September 20, 2020, there were 30,675,675 confirmed cases and 954,417 deaths [1]. Since immunization against COVID-19 is not yet available, the current means for pandemic control is to avoid exposure. These measures (eg, physical distancing and lockdown) are likely detrimental to the global economy. Except for China, all G20 countries, a group of the world’s largest economies, experienced a decrease in gross domestic product in the second quarter of 2020 due to the COVID-19 pandemic [2]. Moreover, implementation of these measures also results in substantial impairment in physical and psychological well-being [3]. There is a strong need for an effective vaccine to keep COVID-19 under control.

Development of the COVID-19 vaccines is on the way. According to the World Health Organization, there are 34 and 142 candidate vaccines in clinical and preclinical evaluation, respectively, as of September 3, 2020; four Chinese candidate vaccines have entered phase III clinical trials [4]. The interim analysis of the phase III trials of a China candidate COVID-19 vaccine (Beijing Institute of Biological Product’s inactivated vaccine Sinopharm) showed that it had 86% vaccine efficacy against COVID-19 [5]. The analysis also showed the vaccine had a 99% seroconversion rate of neutralizing antibodies and a 100% effectiveness in preventing moderate and severe cases of the disease with no serious safety concern [5]. The United Arab Emirates Ministry of Health and Prevention announced the official registration of the vaccine on December 9, 2020 [5]. On July 22, 2020, the National Health Commission of the People’s Republic of China authorized the emergency use of the COVID-19 vaccination and provided COVID-19 vaccines to workers, students, and diplomatic personnel who need to travel aboard, as well as health care workers and personnel working for pandemic and border control [6,7]. According to the recent press release, there were 56,000 Chinese people who had received COVID-19 vaccines developed by Sinopharm before travelling aboard. So far, none of them reported a SARS-CoV-2 infection [8]. It was estimated that at least one COVID-19 vaccine would become available in China by the end of 2020 [9-11]. The market rate will be set at around ¥1000 (about US $154) [10].

The effectiveness of pandemic vaccination campaigns is dependent on both the vaccines’ effectiveness and people’s willingness to be vaccinated. Simulation experiments have shown that, when the reproduction number (R_0_) was 2.5 and vaccination occurred when 5% of the population had been exposed to SARS-CoV-2, a vaccine efficacy of 80% with 75% coverage could reduce the total number of SARS-CoV-2 cases by 85% without any other measures such as social distancing [12]. A systematic review showed that respondents’ willingness to receive a H1N1 influenza A vaccine ranged from 8%-67% [13]. Several factors consistently predict the behavioral intention to receive such a vaccine, including risk of infection, severity of the public health event and personal consequences from the illness, harm or adverse events from the vaccination, use of previous vaccination, and ethnicity [13]. To our knowledge, at least 11 published studies and preprints have investigated behavioral intention to receive a COVID-19 vaccination [14-24]. The impact of COVID-19 on the general population and specific groups is quite different, which may cause different responses to COVID-19 vaccination. For example, health care workers’ risk of COVID-19 infection was 9-11 times higher than the general population [25], and they had the highest priority to receive a COVID-19 vaccination [26]. Free vaccines are likely to be provided to them in the near future. However, prevalence of poor mental health status (eg, depression, anxiety, or posttraumatic stress disorders) caused by a high risk of infection, being overworked, frustration, discrimination, social isolation, and exhaustion were much higher in this group as compared to the general population [27]. This might explain the lower intention to receive COVID-19 vaccination among health care workers (63.0%-76.4%) [23,24] as compared to that of the general population (57.6%-94.3%) [14-17,20-22]. Factory workers who resumed work during the pandemic are another subpopulation at higher risk of COVID-19 infection than that of the general population, as many factories are crowded settings, making physical distancing challenging [28]. COVID-19 outbreaks in the workplace have been reported in China and other countries [28-30]. Moreover, most of the Chinese factory workers are young. Even if infected with COVID-19, many of them may be asymptomatic and unaware of their infection; they may become a driving force of COVID-19 transmission in the workplace and community [31,32]. Previous studies have reported that the prevalence of poor mental health status was similar for factory workers compared to that of the general population [11] and was much lower than health care workers [27]. It is expected that they have a higher motivation to receive a COVID-19 vaccination than the general population and health care workers. COVID-19 vaccination for factory workers is important to achieve a balance of work resumption and pandemic control.

Health promotion is needed even when free vaccinations are available. To develop effective health promotion campaigns, it is important to understand the facilitators and barriers of COVID-19 vaccination uptake among factory workers. Previous studies have found a number of factors associated with behavioral intention to receive a COVID-19 vaccination for the general population or health care workers, including sociodemographics (eg, age, gender, marital status, income, and history of influenza vaccination), presence of comorbid conditions, and trust in government. Moreover, perceptions related to COVID-19 (eg, risk of infection) and COVID-19 vaccination (eg, perceived efficacy, concerns of side effects, other’s acceptance, and confidence to receive vaccination) influenced their intention to receive a COVID-19 vaccination [14-24]. We considered these factors in this study. Theory-based interventions are more effective than those that are not [33]. In this study, we applied the theory of planned behavior (TPB) as the theoretical framework [34]. The TPB postulates that behavioral intention to adopt a health-related behavior (eg, uptake of a COVID-19 vaccination) is a strong predictor of actual behavior. To form such an intention, one would evaluate the pros and cons of the behavior (positive and negative attitudes), consider whether their significant others would support such behavior (perceived subjective norm), and appraise how much control one has over the behavior (perceived behavioral control) [34]. In recent studies, the TPB has been used to successfully explain behavioral intention and actual behaviors to receive human papillomavirus (HPV) and influenza vaccinations [35-37].

Across countries, it is common to encounter vaccination-related information on social media [38]. Previous studies have shown that over 60% of people in the United States used social media as a common source of information related to HPV and influenza vaccinations [39,40]. During the pandemic, people are also actively seeking information about COVID-19 vaccination on different social media platforms [41]. Several studies have reported that social media use would influence perceptions and behaviors related to vaccination. Four studies found a negative influence of social media on users’ perceptions related to vaccination (eg, increase doubt, fear, or barriers for vaccination) [42-45]. Regarding vaccination uptake, women in the United Kingdom who used social media to gather information reported lower pertussis vaccination uptake during pregnancy [46], while positive associations between information exposure through social media and vaccination were found among White and African American adults in the United States [40] and older adults in China [47]. Moreover, different contents related to COVID-19 may have varying effects on personal preventive measures [48]. In this study, we investigated the associations between exposure to different content related to COVID-19 vaccination on social media and behavioral intention to receive a vaccination.

To the best of our knowledge, there have been no studies investigating behavioral intention to receive a COVID-19 vaccination and associated factors among factory workers who resumed work during the COVID-19 pandemic. To address these gaps, this study investigated behavioral intention to receive a self-financed or free COVID-19 vaccination among a sample of factory workers in Shenzhen, China. We examined the effects of factors including sociodemographics, perceptions related to COVID-19 vaccination based on the TPB, exposure to COVID-19 specific information through different media, and COVID-19 preventive measures implemented by individuals and factories.

## Methods

### Study Design

We conducted a cross-sectional closed online survey of 2053 factory workers in Shenzhen, China on September 1-7, 2020. Of the 13 million residents in Shenzhen in 2018, 65.1% were internal migrants and 34.3% were factory workers [49].

### Participants and Data Collection

This study was conducted in the Longhua district of Shenzhen. In Shenzhen, the majority of the factories are located in Longhua. As of 2018, there are over 2000 factories and about one million factory workers in Longhua. Participants were full-time employees of factories in Shenzhen that were 18 years or older. In Shenzhen, factory workers are required to receive a physical examination at designated hospitals or the Centre for Disease Control and Prevention (CDC) annually. All five designated hospitals (three public and two private) and the one district CDC providing physical examination services to factory workers in Longhua were our study sites for recruitment. To avoid selection bias, the fieldworkers approached all adults attending these sites for physical examination during the study period. They briefed prospective participants about the study details, confirmed their eligibility, and invited them to join the study. Participants were guaranteed that participation was voluntary, refusal would have no effect on them, the survey would not collect personal contacts or identification, and data would be kept strictly confidential and only be used for research purposes. Verbal consent was obtained instead of written consent to allow participants to maintain anonymity. We developed an online questionnaire using Questionnaire Star, a commonly used online survey platform in China. Quick Response (QR) codes were generated to access the online questionnaire. Prospective participants were asked to scan the QR code on site to complete the survey. Each mobile device was only allowed to access the online questionnaire once to avoid duplicate responses. The participants were asked not to disseminate the QR codes to access the survey to other people. The survey had 66 items (about 15 items per page for four pages), which took about 15 minutes to complete. The Questionnaire Star performed completeness checks before the questionnaire was submitted. Participants were able to review and change their responses through a “Back” button. An e-coupon of ¥10 (US $1.54) was sent to participants upon completion. In case participants did not have internet access or a smartphone, the research team prepared a tablet computer in each study site for them to complete the online survey. All data was stored in the online server of Questionnaire Star and protected by a password. Only the corresponding author had access to the database. Ethics approval was obtained from the Longhua District CDC (reference: 2020001).

### Measures

#### Design of the Questionnaire

A panel consisting of one CDC staff, two public health researchers, a health psychologist, a senior factory manager, and a factory worker was formed to develop the questionnaire used in this study. The questionnaire was pilot-tested among 10 factory workers to assess clarity and readability. These 10 workers did not participate in the actual survey. Based on the workers’ comments, the panel revised and finalized the questionnaire.

#### Background Characteristics

Participants were asked to report on sociodemographics such as age, gender, relationship status, whether they had a child, highest education level, monthly personal income, status as frontline workers or management, and type of factory they were working in. In addition, participants were also asked about history of seasonal influenza vaccination and whether they had a family member with a history of COVID-19.

#### Behavioral Intention to Receive COVID-19 Vaccination Under Different Scenarios

Participants were briefed with the following: “COVID-19 vaccines developed by China are likely to become available by the end of 2020.” We assessed behavioral intention to receive COVID-19 vaccination under four scenarios: (1) conditional on 50% vaccine efficacy and market rate (¥1000 or US $154), (2) conditional on 80% vaccine efficacy and market rate, (3) conditional on 50% vaccine efficacy and free vaccines, and (4) conditional on 80% vaccine efficacy and free vaccines. On June 2020, the US Food and Drug Administration released guidance for development and licensure of vaccines to prevent COVID-19, which stated that the primary efficacy end point estimate for a placebo-controlled efficacy trial for a COVID-19 vaccine should be at least 50% to ensure that a widely deployed vaccine is effective [50]. Therefore, we chose a threshold of 50% as the lowest estimate of COVID-19 vaccine efficacy in this study. Another study also measured behavioral intention to receive a COVID-19 vaccination conditional on 50% vaccine efficacy [18]. Based on the results of the simulation experiments mentioned in the previous paragraph, Bartsch et al [12] concluded that the vaccine has to have an efficacy of at least 80% to extinguish the COVID-19 epidemic without any other measures. Therefore, we chose a threshold of 80% as an optimal estimation of vaccine efficacy. The threshold of 80% was also close to the vaccine efficacy of the China candidate vaccine (86%) in phase III clinical trials [5]. The cost of COVID-19 vaccines was based on available information in the press release [10].

The response categories were 1 (very unlikely), 2 (unlikely), 3 (neutral), 4 (likely), and 5 (very likely). Behavioral intention was defined as “likely” or “very likely.” This definition has been commonly used in previous studies [51-53]. In this study, we measured behavioral intention to receive a COVID-19 vaccination under the condition that its efficacy and cost was made known to the participants. The process ensured that all participants received uniform information and, hence, allowed for better interpretation of the results.

#### Perceptions Related to COVID-19 Vaccination Based on the TPB

Three scales were constructed to assess perceptions related to COVID-19 vaccination based on the TPB. They were (1) the five-item Positive Attitude Scale (eg, *COVID-19 vaccination is highly effective in protecting you from COVID-19*), (2) the four-item Negative Attitude Scale (eg, *COVID-19 vaccines will have severe side effects*), and (3) the two-item Perceived Subjective Norm Scale (perceived support from doctors/nurses and family members/friends; response categories: 1, disagree; 2, neutral; and 3, agree). The Cronbach α of these scales ranged from .67 to .85; single factors were identified by exploratory factor analysis, explaining for 50.7%-54.0% of total variance. In addition, perceived behavioral control to receive a COVID-19 vaccination was measured by a single item (*receiving a COVID-19 vaccination is easy for you if you want to*; 1, disagree; 2, neutral; and 3, agree).

#### Influence of Social Media

Participants were asked to report the frequency of their exposure to the following information related to a COVID-19 vaccination on social media (WeChat, WeChat moments, Weibo, Tiktok, etc) in the past month (response categories: 1, almost never; 2, seldom; 3, sometimes; 4, always). Such information included positive information related to COVID-19 vaccination (eg, new vaccines entering clinical trials), negative information related to COVID-19 vaccination (eg, concerns about efficacies and supplies), testimonials given by participants of the COVID-19 clinical trials, and negative information about vaccine incidents in China (eg, selling problematic vaccines and severe side effects).

#### COVID-19 Preventive Measures Implemented by Individuals and Factories

Participants were asked to report frequency of wearing face masks when having close contact with others in a workplace and other public settings (public spaces or transportation) in the past month (response categories: every time, often, sometimes, never). Participants also reported frequency of sanitizing hands using soaps, liquid soaps, or alcohol-based hand rubs after returning from public spaces or touching public installations or equipment and whether they avoided social or meal gatherings with people who they do not live with and crowded places in the past month. The Shenzhen government advocated that eight preventive measures should be implemented in the factories, including (1) prohibiting nonemployees from entering workplaces, (2) taking body temperature and sanitizing hands for all employees before entering the workplace, (3) providing face masks to all employees, (4) keeping adequate distance (eg, >1 meter) between workstations, (5) requiring employees to wear face masks when they have close contact with other people, (6) disinfecting the workplace frequently, (7) maintaining adequate ventilation in the workplace, and (8) setting up partitions in factory canteens [54,55]. Participants reported whether their factory implemented these eight preventive measures. A composite indicator variable was constructed by counting the number of preventive measures implemented by the factory (ranging from 0 to 8). The English and Chinese versions of the questionnaire are shown in Multimedia Appendix 1.

### Sample Size Planning

The target sample size was 2000. Given a statistical power of 0.80 and an alpha value of .05, and assuming the level of behavioral intention to receive a COVID-19 vaccination in the reference group (without a facilitating condition) to be 30%-70%, the sample size could detect a smallest odds ratio (OR) of 1.29 between those with and without such facilitating condition (PASS 11.0; NCSS, LLC).

### Statistical Analysis

The binary variables on behavioral intention to receive a COVID-19 vaccination conditional on 80% vaccine efficacy and market rate and conditional on same efficacy and free vaccines were used as the dependent variables. Multilevel logistic regression models (level 1: study sites; level 2: individual participants) were used to analyze factors associated with the dependent variables. Random intercept models were used to allow the intercept of the regression model to vary across study sites, which could account for intracorrelated nested data. Multilevel logistic regression models are commonly used in studies with similar sampling methods [28,56]. A univariate two-level logistic regression model first assessed the significance of the association between each of the background characteristics and the dependent variables. Background characteristics with *P*<.05 in univariate analysis were adjusted in the multivariate two-level logistic regression model.

In addition, using behavioral intention to receive a COVID-19 vaccination conditional on 80% vaccine efficacy and market rate and conditional on same efficacy and free vaccines were used as ordinal dependent variables (from 1 to 5), and background characteristics were used as independent variables; ORs were obtained using ordinal logistic regressions. Adjustment for significant background characteristics, associations between independent variables of interest (perceptions, information exposure through social media, and preventive measures implemented by individuals and factories) and the dependent variables were then assessed by adjusted odds ratios (AORs). A similar approach was used in previous studies [57]. Principal component analysis with varimax rotation was used to perform explanatory factor analysis. Correlations between information exposure through social media and perceptions related to COVID-19 vaccination were also investigated. Pearson correlation coefficients (*r*) were obtained. SPSS version 26.0 (IBM Corp) was used for data analysis, with *P*<.05 considered statistically significant.

## Results

### Background Characteristics

Out of 2653 eligible factory workers (between 60 and 1200 across study sites) that were approached, 2053 completed the online survey (between 40 and 968 across study sites). The overall response rate was 77.4% (ranging from 66.7% to 80.7% at different sites). Main reasons for nonresponse were lack of time and other logistic reasons. All participants that were approached had access to the internet or a smartphone, and none of them used the tablet computers prepared by the research team. Over half of the participants were younger than 40 years (n=1490, 72.6%), were female (n=1179, 57.4%), were married (n=1455, 70.9%), had children (n=1466, 71.4%), did not receive tertiary education (n=1472, 71.7%), had a monthly income less than ¥5000 (US $773; n=1421, 69.2%), were frontline workers (n=1476, 71.9%), and were working for electronic device manufacturers (n=1473, 71.7%). Among the participants, 20.3% (n=416) had received a seasonal influenza vaccination at least once, and 1.6% (n=32) had at least one family member with a history of COVID-19 (Table 1).

**Table 1:** Background characteristics of the participants (N=2053).

Characteristics		Participants, n (%)
**Age group (years)**		
	18-30	757 (36.9)
	31-40	733 (35.7)
	41-50	491 (23.9)
	>50	72 (3.5)
**Gender**		
	Male	874 (42.6)
	Female	1179 (57.4)
**Relationships status**		
	Currently single	448 (21.8)
	Having a stable boyfriend/girlfriend	150 (7.3)
	Married	1455 (70.9)
**Have children**		
	No	587 (28.6)
	Yes	1466 (71.4)
**Highest education level attained**		
	Junior high or below	859 (41.8)
	Senior high or equivalent	613 (29.9)
	College/university or above	581 (28.3)
**Monthly personal income (¥; US $)**		
	<3000 (463.84)	512 (24.9)
	3000-4999 (463.84-772.92)	909 (44.3)
	5000-6999 (773.07-1082.15)	359 (17.5)
	7000-9999 (1082.30-1545.99)	150 (7.3)
	≥10,000 (1546.14)	123 (6.0)
**Type of work**		
	Frontline workers	1476 (71.9)
	Management staff	577 (28.1)
**Factory type**		
	Electronic devices manufacturers	1473 (71.7)
	Other factories	580 (28.3)
**History of seasonal influenza vaccination**		
	No	1637 (79.7)
	Yes	416 (20.3)
**Having at least one family member with a history of COVID-19**		
	No	2021 (98.4)
	Yes	32 (1.6)

### Behavioral Intention to Receive COVID-19 Vaccination Under Different Scenarios

The prevalence of behavioral intention to receive a COVID-19 vaccination was 53.5% (n=1099, conditional on 50% vaccine efficacy and market rate), 66.6% (n=1368, conditional on 80% vaccine efficacy and market rate), 75.6% (n=1551, conditional on 50% vaccine efficacy and free vaccines), and 80.6% (n=1655, conditional on 80% vaccine efficacy and free vaccines; Table 2).

**Table 2:** Perceptions related to COVID-19 vaccination and preventive measures taken up by participants and the factories they were working in (N=2053).

Perceptions			Participants, n (%)		Mean (SD)	
**Behavioral intention to take up COVID-19 vaccination**						
	**Intention to get COVID-19 vaccines conditional on 50% efficacy and market rate (¥1000 or US $140)**					N/A^a^
		Very unlikely		222 (10.8)		
		Unlikely		208 (10.1)		
		Neutral		524 (25.5)		
		Likely		543 (26.4)		
		Very likely		556 (27.1)		
	**Intention to get COVID-19 vaccines conditional on 80% efficacy and market rate (¥1000 or US $140)**					N/A
		Very unlikely		155 (7.5)		
		Unlikely		147 (7.2)		
		Neutral		383 (18.7)		
		Likely		639 (31.1)		
		Very likely		729 (35.5)		
	**Intention to get COVID-19 vaccines conditional on 50% efficacy and free vaccines**					N/A
		Very unlikely		108 (5.3)		
		Unlikely		98 (4.8)		
		Neutral		296 (14.4)		
		Likely		453 (22.1)		
		Very likely		1098 (53.5)		
	**Intention to get COVID-19 vaccines conditional on 80% efficacy and free vaccines**					N/A
		Very unlikely		117 (5.7)		
		Unlikely		77 (3.8)		
		Neutral		204 (9.9)		
		Likely		320 (15.6)		
		Very likely		1335 (65.0)		
**Perceptions related to COVID-19 vaccination based on the theory of planned behavior**						
	**Positive attitudes toward COVID-19 vaccination**			N/A		
		Positive Attitude Scale^b^				13.3 (2.3)
	**Negative attitudes toward COVID-19 vaccination**			N/A		
		Negative Attitude Scale^c^				8.0 (1.9)
	**Perceived subjective norm related to COVID-19 vaccination**			N/A		
		Perceived Subjective Norm Scale^d^				5.1 (1.2)
	**Perceived behavioral control to receive COVID-19 vaccination**			N/A		
		Receiving COVID-19 vaccination is easy for you if you want to				2.3 (0.7)
**Influence of social media related to COVID-19 vaccination**						
	Frequency of exposure to positive information related to COVID-19 vaccination (eg, new vaccines entering clinical trials, promising efficacies of the vaccines, and vaccines will enter the market soon) on social media			N/A		2.8 (1.0)
	Frequency of exposure to negative information related to COVID-19 vaccination (eg, concerns about efficacies and supplies, side effects of the vaccines, and receiving vaccines will cause COVID-19) on social media			N/A		2.3 (0.9)
	Frequency of exposure to testimonials given by participants of the COVID-19 vaccine clinical trials on social media			N/A		1.9 (1.0)
	Frequency of exposure to negative information about other vaccines in China (eg, selling problematic vaccines and severe side effects) on social media			N/A		2.0 (1.0)
**Personal COVID-19 preventive measures in the past month**						
	**Frequency of wearing a face mask in public places/transportation other than the workplace**					N/A
		Every time		1675 (81.6)		
		Often		280 (13.6)		
		Sometimes		82 (4.0)		
		Never		16 (0.8)		
	**Frequency of wearing a face mask when you have close contact with other people in the workplace**					N/A
		Every time		1519 (74.0)		
		Often		370 (18.0)		
		Sometimes		144 (7.0)		
		Never		20 (1.0)		
	**Self-reported sanitizing hands (using soaps, liquid soaps, or alcohol-based sanitizer) after returning from public spaces or touching public installation**					N/A
		Every time		1217 (59.3)		
		Often		495 (24.1)		
		Sometimes		323 (15.7)		
		Never		18 (0.9)		
	**Self-reported avoiding social gatherings with other people who do not live together**					N/A
		No		1165 (56.7)		
		Yes		888 (43.3)		
	**Self-reported avoiding crowded places**					N/A
		No		1309 (63.8)		
		Yes		744 (36.2)		
Number of preventive measures implemented by the factory			N/A		6.1 (2.5)	

^a^N/A: not applicable.

^b^Positive Attitude Scale, 5 items, Cronbach alpha: .84; 1 factor was identified by exploratory factor analysis, explaining for 54.0% of total variance.

^c^Negative Attitude Scale, 4 items, Cronbach alpha: .67; 1 factor was identified by exploratory factor analysis, explaining for 50.9% of total variance.

^d^Perceived Subjective Norm Scale, 2 items, Cronbach alpha: .85.

### Perceptions Related to COVID-19 Based on the TPB and Influence of Social Media

Means and SDs of items and scales related to COVID-19 vaccination based on the TPB are described in Table 2 and Multimedia Appendix 2. Among the participants, 66.4% (n=1363) were sometimes or always exposed to positive information related to COVID-19 vaccinations in the past month, while fewer participants were sometimes or always exposed to negative information related to COVID-19 vaccinations (n=842, 41.0%) or other vaccines in China (n=571, 27.8%), or exposed to testimonials given by participants of COVID-19 vaccination clinical trials (n=594, 28.9%).

### COVID-19 Preventive Measures Implemented by Individuals and Factories

In the past month, 74.0% (n=1519) and 81.6% (n=1675) of participants reported wearing a face mask every time they had close contact with other people in the workplace and in other public settings, respectively. More than half of the participants self-reported sanitizing hands (n=1217, 59.3%), avoiding social or meal gatherings (n=1165, 56.7%), and avoiding crowded places (n=1309, 63.8%; Table 2 and Multimedia Appendix 2).

### Factors Associated With Behavioral Intention to Receive a COVID-19 Vaccination

In the univariate logistic regression analysis, age group, relationship status, having children, education level, monthly personal income, status as frontline workers or management staff, history of seasonal influenza vaccination, and having a family member with a history of COVID-19 were significantly associated with one or both dependent variables (Table 3).

**Table 3:** Associations between background characteristics and behavioral intention to receive COVID-19 vaccination under different scenarios (N=2053).

Characteristics		Conditional on 80% efficacy and market rate, OR^a,b^ (95% CI)	Conditional on 80% efficacy and free vaccines, OR (95% CI)
**Age group (years)**			
	18-30	1.0	1.0
	31-40	0.80 (0.64-1.01)	0.95 (0.72-1.25)
	41-50	0.62 (0.48-0.79)^***^	0.61 (0.42-0.76)^***^
	>50	0.48 (0.29-0.79)^**^	0.57 (0.32-1.02)
**Gender**			
	Male	1.0	1.0
	Female	1.16 (0.96-1.41)	0.99 (0.78-1.24)
**Relationships status**			
	Currently single	1.0	1.0
	Having a stable boyfriend/girlfriend	1.44 (0.94-2.20)	1.55 (0.89-2.71)
	Married	0.94 (0.74-1.18)	0.77 (0.58-1.02)
**Having children**			
	No	1.0	1.0
	Yes	0.77 (0.62-0.95)^*^	0.66 (0.50-0.86)^*^
**Highest education level attained**			
	Junior high or below	1.0	1.0
	Senior high or equivalent	1.57 (1.26-1.96)^***^	2.09 (1.60-2.73)^***^
	College/university or above	1.94 (1.52-2.47)^***^	3.39 (2.69-5.01)^***^
**Monthly personal income (¥; US $)**			
	<3000 (463.84)	1.0	1.0
	3000-4999 (463.84-772.92)	1.11 (0.89-1.40)	1.20 (0.89-1.50)
	5000-6999 (773.07-1082.15)	1.36 (1.01-1.83)^*^	1.39 (0.99-1.97)^*^
	7000-9999 (1082.30-1545.99)	1.47 (0.97-2.21)^*^	1.76 (1.05-2.94)^*^
	≥10,000 (1546.14)	1.60 (1.01-2.56)^*^	4.82 (2.05-11.36)^***^
**Type of work**			
	Frontline workers	1.0	1.0
	Management staff	1.24 (1.01-1.54)^**^	1.66 (1.26-2.19)^***^
**Factory type**			
	Electronic devices manufacturer	1.0	1.0
	Other factories	1.27 (0.97-1.52)	1.09 (0.83-1.42)
**History of seasonal influenza vaccination**			
	No	1.0	1.0
	Yes	1.29 (1.02-1.64)^*^	1.28 (0.95-1.71)
**Having a family member with history of COVID-19**			
	No	1.0	1.0
	Yes	4.85 (2.21-10.53)^***^	6.49 (3.10-13.51)^***^

^a^OR: odds ratio.

^b^Crude ORs obtained by using univariate two-level logistic regression models.

^*^P<.05, ^**^P<.01, ^***^P<.001.

After adjusting for these significant background characteristics, positive attitudes toward COVID-19 vaccination (AOR 1.20, 95% CI 1.15-1.25 and AOR 1.24, 95% CI 1.19-1.30), perceived support from significant others on COVID-19 vaccination uptake (AOR 1.43, 95% CI 1.32-1.55 and AOR 1.37, 95% CI 1.25-1.50), and perceived behavioral control to get a COVID-19 vaccination (AOR 1.51, 95% CI 1.32-1.73 and AOR 1.28, 95% CI 1.09-1.51) were positively associated with both dependent variables (dependent on 80% efficacy and market rate vaccines and dependent on 80% efficacy and free vaccines, respectively). Regarding social media influence, higher frequency of exposure to positive information related to a COVID-19 vaccination was associated with higher intention to receive a COVID-19 vaccination at market rate (AOR 1.53, 95% CI 1.39-1.70) or to receive a free vaccination (AOR 1.52, 95% CI 1.35-1.71). Higher self-reported compliance with wearing a face mask in the workplace (AOR 1.27, 95% CI 1.02-1.58 and AOR 1.67, 95% CI 1.24-2.27) and other public spaces (AOR 1.80, 95% CI 1.42-2.29 and AOR 1.34, 95% CI 1.01-1.77), hand hygiene (AOR 1.21, 95% CI 1.00-1.47 and AOR 1.52, 95% CI 1.19-1.93), and avoiding social and meal gatherings (AOR 1.22, 95% CI 1.01-1.47 and AOR 1.55, 95% CI 1.23-1.95) and crowded places (AOR 1.24, 95% CI 1.02-1.51 and AOR 1.73, 95% CI 1.37-2.18) were also positively associated with one or both dependent variables (dependent on 80% efficacy and market rate vaccines and dependent on 80% efficacy and free vaccines, respectively). A higher number of COVID-19 preventive measures implemented by the factory were significantly associated with a higher intention to receive COVID-19 vaccination under both scenarios (AOR 1.08, 95% CI 1.04-1.12 and AOR 1.06, 95% CI 1.01-1.11, respectively; Table 4).

**Table 4:** Factors associated with behavioral intention to receive a COVID-19 vaccination under different scenarios (N=2053).

Factors			Conditional on 80% and market rate, AOR^a,b^ (95% CI)	Conditional on 80% efficacy and free vaccines, AOR (95% CI)
**Perceptions relate to COVID-19 vaccination based on the theory of planned behavior**				
	Positive Attitude Scale		1.20 (1.15-1.25)^***^	1.24 (1.19-1.30)^***^
	Negative Attitude Scale		0.98 (0.93-1.03)	1.00 (0.94-1.06)
	Perceived Subjective Norm Scale		1.43 (1.32-1.55)^***^	1.37 (1.25-1.50)^***^
	Perceived behavioral control to receive COVID-19 vaccination		1.51 (1.32-1.73)^***^	1.28 (1.09-1.51)^**^
**Influence of social media related to COVID-19 vaccination**				
	Frequency of exposure to positive information related to COVID-19 vaccination on social media		1.53 (1.39-1.70)^**^	1.52 (1.35-1.71)^***^
	Frequency of exposure to negative information related to COVID-19 vaccination on social media		1.11 (0.99-1.23)	1.07 (0.95-1.21)
	Frequency of exposure to testimonials given by participants of the COVID-19 vaccine clinical trials on social media		1.10 (0.99-1.21)	1.00 (0.89-1.11)
	Frequency of exposure to negative information about vaccine incidents in China on social media		0.95 (0.86-1.04)	0.93 (0.83-1.05)
**Personal COVID-19 preventive measures in the past month**				
	**Consistent use of face mask in public places/transportation other than the workplace**			
		No	1.0	1.0
		Yes	1.80 (1.42-2.29)^***^	1.34 (1.01-1.77)^*^
	**Consistent use of face mask when you have close contact with other people in the workplace**			
		No	1.0	1.0
		Yes	1.27 (1.02-1.58)^*^	1.67 (1.24-2.27)^**^
	**Self-reported sanitizing hands (using soaps, liquid soaps, or alcohol-based sanitizer) every time after returning from public spaces or touching public installation**			
		No	1.0	1.0
		Yes	1.21 (1.00-1.47)^*^	1.52 (1.19-1.93)^**^
	**Self-reported avoiding social gatherings with other people who do not live together**			
		No	1.0	1.0
		Yes	1.22 (1.01-1.47)^*^	1.55 (1.23-1.95)^***^
	**Self-reported avoiding crowded places**			
		No	1.0	1.0
		Yes	1.24 (1.02-1.51)^*^	1.73 (1.37-2.18)^***^
Number of preventive measures implemented by the factory			1.08 (1.04-1.12)^***^	1.06 (1.01-1.11)^*^

^a^AOR: adjusted odds ratios

^b^Background characteristics with *P*<.05 in univariate two-level logistic regression analysis were adjusted in the multivariate two-level logistic regression models.

^*^P<.05, ^**^P<.01, ^***^P<.001.

When behavioral intention was treated as ordinal variables (from 1 to 5) and used as dependent variables, the same sets of associated factors were identified by univariate and multivariate ordinal logistic regression models. The results are presented in Multimedia Appendix 3.

### Correlation Between Information Exposure Through Social Media and Perceptions Related to COVID-19 Vaccination Based on the TPB

Frequency of exposure to positive information related to COVID-19 vaccinations on social media was positively correlated with positive attitudes (*r*=0.083; *P*<.001), perceived subjective norm (*r*=0.101; *P*<.001), and perceived behavioral control (*r*=0.064; *P*=.004) related to a COVID-19 vaccination. A negative correlation was found between social media exposure and negative attitudes toward a COVID-19 vaccination (*r*=–0.090; *P*<.001). Moreover, frequency of exposure to positive information related to COVID-19 vaccinations on social media was negatively correlated with positive attitudes (*r*=–0.080; *P*<.001), perceived subjective norm (*r*=–0.107; *P*<.001), and perceived behavioral control (*r*=–0.069; *P*=.002) related to a COVID-19 vaccination. Furthermore, frequency of exposure to testimonials given by COVID-19 vaccine clinical trial participants on social media was negatively correlated with positive attitudes (*r*=–0.052, *P*=.02) but was positively correlated with perceived behavioral control to get a COVID-19 vaccination (*r*=0.062, *P*=.005). In addition, frequency of exposure to negative information about other vaccines in China on social media was negatively correlated with positive attitudes (*r*=–0.106, *P*<.001) and perceived subjective norm (*r*=–0.132, *P*<.001) related to a COVID-19 vaccination (Multimedia Appendix 4).

## Discussion

Our findings represent one of the latest estimates of COVID-19 vaccination acceptability in China and can be used to project future vaccine uptake. Factory workers’ behavioral intention to receive a COVID-19 vaccination was more sensitive to its cost than vaccine efficacy. Given the same vaccine efficacy, the behavioral intention varied from 53.6%-66.6% at market rate to about 80% for a free vaccination. However, small increases were observed (13.1% under market rate and 2% for free vaccination) when comparing behavioral intention conditional on 50% vaccine efficacy and those conditional on 80% vaccine efficacy. The prevalence of behavioral intention conditional on free vaccination was higher than that reported in the United States [16] and Saudi Arabia [22] but was lower than that of the general population in Malaysia [14] and China [23]. A meta-analysis showed that only 43%-62% of those with a behavioral intention would translate that into related actions [58]. Therefore, effective health promotion is needed when COVID-19 vaccines become available to achieve high coverage at a population level.

Our findings provide empirical insights to inform health promotion development. Similar strategies can be used to promote COVID-19 vaccinations at market rate or free vaccinations, as the associated factors of these two dependent variables were similar. Older participants had a lower intention to receive a COVID-19 vaccination. This finding is consistent with a previous study targeting the general population in the United States [20]. Younger people may be more receptive to innovations [59]. Participants with lower education had a lower intention to receive a COVID-19 vaccination compared to workers with higher levels of education. The former might find it difficult to understand information related to COVID-19 vaccination due to a lower literacy level. The finding for income coincides with previous studies that suggested that intention to receive COVID-19 vaccines and uptake of other vaccines were lower among individuals with lower income [20,60]. As compared to frontline workers, management staff were more willing to receive a COVID-19 vaccination. Studies among factory workers have shown that management staff were more likely to adopt personal measures to prevent COVID-19 [28]. Having children was associated with a lower intention to receive a COVID-19 vaccination; future studies should explore whether there are any specific barriers for parents. A history of seasonal influenza vaccination was associated with a higher intention to receive a COVID-19 vaccination at market rate but not with the intention to receive a free vaccination. In China, free seasonal influenza vaccination is provided to older adults, while out-of-pocket payment is required for other groups. Factory workers who had taken a seasonal influenza vaccination may have positive beliefs toward self-financed vaccination. Furthermore, having a family member with history of COVID-19 was strongly associated with a higher intention to receive a COVID-19 vaccination. This finding was expected, as these participants had direct experience related to COVID-19 and were more likely to perceive it as a serious health threat.

The TPB is a potentially useful framework to guide the development of future programs, as three of the four TPB constructs used in this study were significantly associated with both dependent variables in expected directions. It would be useful to increase positive attitudes toward COVID-19 vaccination, as this was found to be a facilitator. In addition to the beneficial effect for oneself (eg, preventing COVID-19 and returning to normal lives), health communication messages should also emphasize that COVID-19 vaccination uptake would result in herd immunization, which could contribute to COVID-19 control. Building up confidence related to the vaccine supply may also be a useful strategy. Over 60% of participants perceived that medical professionals, family, and friends would support them in taking the COVID-19 vaccination. This perception was also a facilitator. Future programs should consider involving the significant others of factory workers to create a subjective norm favoring COVID-19 vaccination uptake. It would also be useful to enhance perceived behavioral control, as this was another facilitator. There is more room for improvement. Outreach in the factories and providing vaccination on-site may be a useful strategy to improve perceived behavioral control among the workers. Relatively few participants had concerns related to cost, side effects, and duration of vaccine protection. The associations between these concerns and behavioral intention were not statistically significant. Addressing these concerns might not be a useful strategy in future promotion campaigns.

Our findings suggest that COVID-19 vaccination triggered intensive responses on social media, as about 60% of the participants sometimes or always were exposed to information specific to COVID-19 vaccination on different social media platforms. Our results showed that exposure to positive information related to COVID-19 vaccination through social media was positively correlated with positive perceptions (ie, positive attitudes, perceived subjective norm, and perceived behavioral control) related to COVID-19 vaccination. These positive perceptions were determinants of behavioral intention to get a COVID-19 vaccination in this study. It is possible that higher amounts of positive information exposure on social media would enhance these positive perceptions, which in turn increases behavioral intention to get a COVID-19 vaccination. Longitudinal studies are needed to test whether this pathway exists. Negative information about vaccines is uniquely attractive to social media. Studies have shown that a major vaccine incident (Changchun Changsheng) had significantly impaired the confidence of vaccines among Chinese people [61]. However in this study, negative information about these vaccine incidents did not influence participants’ behavioral intentions to receive a COVID-19 vaccination.

Factory workers who reported higher compliance to personal preventive measures were more willing to receive a COVID-19 vaccination. These people may have stronger motivation and self-efficacy to protect themselves, and a COVID-19 vaccination is likely to be considered as a useful means for protection. Preventive measures implemented by the factories also played important roles. More measures implemented by the factories was associated with a higher intention to receive a COVID-19 vaccination. Through implementation of these measures, factories could cultivate widely shared organizational norms to facilitate behavioral changes among the workers [28,62].

This study has some limitations. First, a direct measure of perceived behavioral control should assess self-efficacy and perceived controllability [63]. Previous studies have suggested these two constructs were differentially associated with behavioral intention and actual behaviors [63]. Due to the limited length of the questionnaire, we only used a single item to measure perceived behavioral control, which mainly covered self-efficacy. Failure to measure perceived controllability together with self-efficacy was one major limitation of this study. This limitation made this study less comparable to other studies using the TPB. Second, this study focused on factory workers and did not study the general population in Shenzhen. In addition, we only included factory workers in one Chinese city. Generalizations should be made cautiously to individuals working in other places in China. Third, since the study was anonymous and did not collect participants’ identification, we were not able to collect the information of those who refused to join the study. Factory workers who refused to join the study might have different characteristics as compared to study participants. Since most of the factory workers in Shenzhen are internal migrants, these is no accurate census data for this group. Therefore, we were not able to perform weighting for our sample; selection bias existed. However, our response rate was relatively high as compared to other online surveys of similar topics [23]. Fourth, data was self-reported and verification was not feasible. Recall bias might exist. Participants may have also overreported their intention and compliance with personal preventive measures due to social desirability. Fifth, most items and scales used in this study were self-constructed based on those from previous studies on H1N1 and seasonal influenza vaccination in China [64,65]. The internal reliability of these scales were acceptable, but these scales may require external validation. Moreover, casual relationships cannot be determined due to the cross-sectional design of this study.

In summary, factory workers in China reported a high behavioral intention to receive a COVID-19 vaccination. The behavioral intention was cost-sensitive, and the proposed market rate was accepted by the majority of the participants. The TPB is a useful framework to guide the development of future campaigns promoting COVID-19 vaccination in this group.

## Appendix

Multimedia Appendix 1 The English and Chinese versions of the questionnaire used in the online survey.

Multimedia Appendix 2 Item responses of perceptions related to COVID-19 vaccination and preventive measures taken up by participants and the factories they were working in.

Multimedia Appendix 3 Factors associated with behavioral intention to receive a COVID-19 vaccination obtained by using univariate and multivariate ordinal logistic regression models.

Multimedia Appendix 4 Correlations between information exposure through social media and perceptions related to COVID-19 vaccination based on the theory of planned behavior.

## Abbreviations

AOR: adjusted odds ratio
CDC: Centre for Disease Control and Prevention
HPV: human papillomavirus
OR: odds ratio
QR: Quick Response
TPB: theory of planned behavior
