# Supplementary material for: Behavioral Intention to Receive a COVID-19 Vaccination Among Chinese Factory Workers: Cross-sectional Online Survey
Source: J Med Internet Res. 2021 Mar 9;23(3):e24673. doi: 10.2196/24673 (PMC7945977; doi:10.2196/24673)
Supplement: Multimedia Appendix 1 [file jmir_v23i3e24673_app1.docx]

Multimedia Appendix 1

Questionnaire in English and Chinese

**第一部分：对新冠肺炎和疫苗的看法**

**Part 1: Perceptions related to COVID-19 vaccination**

1-1国产新冠肺炎疫苗可能在今年年底上市

COVID-19 vaccines developed by China are likely to become available by the end of this year

| 请问在以下的情况下你会不会接种新冠肺炎疫苗？  What is your likelihood of receiving COVID-19 vaccination under the following conditions | **非常不可能**  **Very unlikely** | **可能**  **不会**  **Unlikely** | **中立**  **Neutral** | **可能会**  **Likely** | **非常**  **可能**  **Very likely** |
| --- | --- | --- | --- | --- | --- |
| A可以减少你感染新冠肺炎的机会达50%，一共需花费1000元  Conditional on 50% vaccine efficacy and market rate (1,000RMB) | 1 | 2 | 3 | 4 | 5 |
| B可以减少你感染新冠肺炎的机会达80%，一共需花费1000元  Conditional on 80% vaccine efficacy and market rate (1,000RMB) | 1 | 2 | 3 | 4 | 5 |
| C可以减少你感染新冠肺炎的机会达50%，免费提供给你  Conditional on 50% vaccine efficacy and free vaccines | 1 | 2 | 3 | 4 | 5 |
| D可以减少你感染新冠肺炎的机会达80%，免费提供给你  Conditional on 80% vaccine efficacy and free vaccines | 1 | 2 | 3 | 4 | 5 |

1-2你是否同意以下关于新冠肺炎疫苗的说法，以下问题没有正确或错误之分，请根据自己的想法作答

Do you agree with the following statements related to COVID-19 vaccination? There is no right or wrong answers.

|  | **不同意**  **Disagree** | **中立**  **Neutral** | **同意**  **Agree** |
| --- | --- | --- | --- |
| A新冠肺炎疫苗可以有效预防你感染新冠肺炎  COVID-19 vaccination is highly effective in protecting you from COVID-19 | 1 | 2 | 3 |
| B接种新冠肺炎疫苗可以保护你的家人不得新冠肺炎  Taking up COVID-19 vaccination is highly effective in protecting your family members against COVID-19 | 1 | 2 | 3 |
| C接种新冠肺炎疫苗可以可以让你的生活回到疫情前的状态  Taking up COVID-19 vaccination can bring your life back to the time before COVID-19 | 1 | 2 | 3 |
| D接种新冠肺炎疫苗可以能为我国的疫情防控作出贡献  Taking up COVID-19 vaccination can contribute to the control of COVID-19 in China | 1 | 2 | 3 |
| E我国可以保证新冠肺炎疫苗供应充足  China will have adequate supply of COVID-19 vaccination | 1 | 2 | 3 |

1-3你同意以下的说法吗？

Do you agree with the following statements?

|  | **不同意**  **Disagree** | **中立**  **Neutral** | **同意**  **Agree** |
| --- | --- | --- | --- |
| A新冠肺炎疫苗可能有严重的副作用  COVID-19 vaccines will have severe side-effects | 1 | 2 | 3 |
| B新冠肺炎疫苗对你的保护作用只能持续较短的时间  The protection of COVID-19 vaccines will only last for a short time | 1 | 2 | 3 |
| C你可能需要经常接种新冠疫苗  You have to receive COVID-19 vaccination frequently | 1 | 2 | 3 |
| D你觉得接种新冠肺炎疫苗的费用很昂贵  The cost of COVID-19 vaccination is expensive for you | 1 | 2 | 3 |
| E如果新冠肺炎疫苗上市，医生护士会支持你接种  Doctors and nurse would support you to receive COVID-19 vaccination | 1 | 2 | 3 |
| F如果新冠肺炎疫苗上市，家人或朋友会支持你接种  Your family members and friends will support you to receive COVID-19 vaccination | 1 | 2 | 3 |
| G如果你想，接种新冠肺炎疫苗对于你来说是件容易的事情  Receiving COVID-19 vaccination is easy for you if you want to | 1 | 2 | 3 |

1-7请问你在过去一个月内在社交媒体（朋友圈、微博、抖音等）上有多经常看到以下的内容

Frequency of exposing to the following information related to COVID-19 vaccination on social media (WeChat, WeChat moments, Weibo, Tiktok, etc.) in the past month

|  | **几乎**  **没有**  **Almost never** | **很少**  **Seldom** | **有时**  **Sometimes** | **经常**  **Always** |
| --- | --- | --- | --- | --- |
| A 新冠疫苗有关的积极信息（例如疫苗进入临床试验、疫苗的效果、疫苗即将上市）  Positive information related to COVID-19 vaccination (e.g., new vaccines entering clinical trials, promising efficacy of the vaccines, and vaccines, and vaccines will enter the market soon) | 0 | 1 | 2 | 3 |
| B新冠疫苗有关的负面信息（例如对疫苗效果的质疑、疫苗的副作用、对供应的担忧、接种疫苗也会得新冠等等）  Negative information related to COVID-19 vaccination (e.g., concerns about efficacies and supplies, side-effects of the vaccines, and receiving vaccines will cause COVID-19) | 0 | 1 | 2 | 3 |
| C接种了新冠疫苗的人分享自己接种后的感觉  Testimonials given by participants of the COVID-19 vaccine clinical trials | 0 | 1 | 2 | 3 |
| D国产其他疫苗的负面消息（例如制造销售不合格或过期疫苗、接种疫苗导致严重副作用）  Negative information about other vaccines in China (e.g., selling problematic vaccines and severe side effects) | 0 | 1 | 2 | 3 |

**第二部分：防疫行为**

**Part 2: Preventive measures**

**个人防疫行为**

**Personal preventive measures**

2-1过去一个月内，你在公众场所或者搭乘公共交通工具时是否佩戴口罩?

Frequency of facemask wearing in public places/transportations other than workplaces in the past month

□_1_每次都佩戴 Every time □_2_ 经常佩戴 Often

□_3_ 有时佩戴 Sometimes □_4_从不佩戴 Never

2-2过去一个月内，在上班的过程中，当你与他人有近距离接触是否佩戴口罩？

Frequency of facemask wearing when you have close contact with other people in workplace in the past month

□_1_每次都佩戴 Every time □_2_ 经常佩戴 Often

□_3_ 有时佩戴 Sometimes □_4_从不佩戴 Never

2-3过去一个月内，你去过公共地方或接触公共设施后有没有用肥皂/洗手液洗手或者消毒双手？

Frequency of sanitizing hands (using soaps, liquid soaps or alcohol-based sanitizer) after returning from public spaces or touching public installation

□_1_每次都用 Every time □_2_ 经常用 Often

□_3_ 有时用 Sometimes □_4_从不 Never

| 2-4在过去一个月内，你有没有……  In the past month, did you…… | **有**  **Yes** | **没有**  **No** |
| --- | --- | --- |
| A避免与非同住的人聚会/聚餐  Avoid social/meal gathering with other people who do not live together | 1 | 2 |
| B避免到人多的地方  Avoid crowed places | 1 | 2 |

**企业防疫行为**

Preventive measures implemented by workplace

| 2-5据您所知，你所在的企业**现在**是否采取了以下的措施  To your knowledge, did your workplace implement the following preventive measures? | **有**  **Yes** | **没有**  **No** |
| --- | --- | --- |
| A禁止外卖、快递等无关人员进入工作场所  Prohibiting non-employees entering workplace | 1 | 2 |
| B为所有进入工作场所的员工量体温和消毒双手  Taking body temperature and sanitizing hands for all employees entering the workplace | 1 | 2 |
| C为所有员工提供口罩  Providing facemasks to all employees | 1 | 2 |
| D保证工位之间的间隔（例如至少1米）  Keeping adequate distance (e.g., >1m) between work stations | 1 | 2 |
| E上班期间，要求员工与他人近距离接触时佩戴口罩  Requiring employees to wear facemasks when they have close contact with other people | 1 | 2 |
| F为工作场所经常消毒  Frequent workplace disinfection | 1 | 2 |
| G保持工作场所通风良好  Maintaining adequate ventilation in workplace | 1 | 2 |
| H就餐位置之间设置隔板  Setting up partitions in factory canteens | 1 | 2 |

**最后一部分：社会人口学特征**

**Last part: Socio-demographics**

3-1您的年龄是： _______岁

How old are you: _____ years

3-2您的性别为: □_1_男 □_2_ 女

What is your gender? □_1_Male □_2_ Female

3-3请问您目前的婚姻状况是：

What is your relationship status?

□_1_ 未婚且没有固定的男/女朋友 Without a stable boyfriend/girlfriend

□_2_ 未婚但是有固定的男/女朋友 With a stable boyfriend/girlfriend

□_3_ 已婚 Married

□_4_ 离婚/丧偶 Divorced/widowed

3-4请问您的学历是：

What is your education level?

□_1_ 小学及以下 Primary school or below

□_2_ 初中 Junior high

□_3_ 高中/中专 Senior high or equivalent

□_4_ 大专 College

□_5_ 本科 University

□_6_ 硕士及以上 Postgraduate

3-5请问您目前的平均月收入是：

What is your monthly income level?

□_1_ 1000元以下 Below 1000RMB

□_2_ 1000-2999元 1000-2999 RMB

□_3_ 3000-4999元 3000-4999 RMB

□_4_ 5000-6999元 5000-6999 RMB

□_5_ 7000-9999元 7000-9999 RMB

□_6_ 10000元及以上 10,000 RMB or above

□_7_ 无固定收入 No fixed income

3-8您目前所从事的职业角色

Are you a frontline worker or a management staff?

□_1_工人 frontline worker □_2_管理层 management staff

3-9请问您有没有接种过流感疫苗？

Have you ever received seasonal influenza vaccination?

□_1_ 没有 No

□_2_ 一年以内接种过 Yes, within one year

□_3_ 超过一年前接种过 Yes, beyond one year ago

3-10请问您是否有家人感染了新冠肺炎？

Do you have a family member with history of COVID-19?

□_1_有 Yes □_2_没有 No

3-11请问你有没有子女？

Do you have any child?

□_1_没有子女 No □_2_有子女，都已经成年 Yes, all of them are at least 18 years old

□_3_有子女，其中有至少一位未成年 Yes, at least one of them is under the age of 18 years
