# Supplementary material for: Behavioral Intention to Receive a COVID-19 Vaccination Among Chinese Factory Workers: Cross-sectional Online Survey
Source: J Med Internet Res. 2021 Mar 9;23(3):e24673. doi: 10.2196/24673 (PMC7945977; doi:10.2196/24673)
Supplement: Multimedia Appendix 2 [file jmir_v23i3e24673_app2.docx]

Multimedia Appendix 2

Table 1 Items response of perceptions related to COVID-19 vaccination and preventive measures taken up by participants and the factories they were working (n=2053)

|  | N (%) |
| --- | --- |
| **Perceptions relate to COVID-19 vaccination based on the Theory of Planned Behavior** |  |
| Positive attitudes toward COVID-19 vaccination (% agree) |  |
| COVID-19 vaccination is highly effective in protecting you from COVID-19 | 1505 (73.3) |
| Taking up COVID-19 vaccination is highly effective in protecting your family members against COVID-19 | 1477 (71.9) |
| Taking up COVID-19 vaccination can bring your life back to the time before COVID-19 | 1287 (62.7) |
| Taking up COVID-19 can contribute to the control of COVID-19 in China | 1703 (83.0) |
| China will have adequate supply of COVID-19 vaccination | 1437 (70.0) |
|  |  |
| Negative attitudes toward COVID-19 vaccination (% agree) |  |
| COVID-19 vaccines will have severe side-effects | 309 (15.1) |
| The protection of COVID-19 vaccines will only last for a short time | 439 (21.4) |
| You have to receive COVID-19 vaccination frequently | 540 (26.3) |
| The cost of COVID-19 vaccination is expensive for you | 681 (33.2) |
|  |  |
| Perceived subjective norm related to COVID-19 vaccination (% agree) |  |
| Doctors and nurses would support you to receive COVID-19 vaccination | 1270 (61.9) |
| Your family members and friends will support you to receive COVID-19 vaccination | 1260 (61.4) |
|  |  |
| Perceived behavioral control to receive COVID-19 vaccination (% agree) |  |
| Receiving COVID-19 vaccination is easy for you if you want to | 845 (41.2) |
|  |  |
| **Influence of social media related to COVID-19 vaccination** |  |
| Frequency of exposure to positive information related to COVID-19 vaccination (e.g., new vaccines entering clinical trials, promising efficacies of the vaccines, and vaccines will enter the market soon) on social media |  |
| Almost never | 223 (11.5) |
| Seldom | 454 (22.1) |
| Sometimes | 783 (38.1) |
| Always | 580 (28.3) |
| Frequency of exposure to negative information related to COVID-19 vaccination (e.g., concerns about efficacies and supplies, side-effects of the vaccines, and receiving vaccines will cause COVID-19) on social media |  |
| Almost never | 487 (23.7) |
| Seldom | 724 (35.3) |
| Sometimes | 622 (30.3) |
| Always | 220 (10.7) |
| Frequency of exposure to testimonials given by participants of the COVID-19 vaccine clinical trials on social media |  |
| Almost never | 911 (44.4) |
| Seldom | 548 (26.7) |
| Sometimes | 394 (19.2) |
| Always | 200 (9.7) |
| Frequency of exposure to negative information about other vaccines in China (e.g., selling problematic vaccines and severe side-effects) on social media |  |
| Almost never | 834 (40.6) |
| Seldom | 648 (31.6) |
| Sometimes | 399 (19.4) |
| Always | 172 (8.4) |
|  |  |
| **Personal COVID-19 preventive measures in the past month** |  |
| Frequency of face mask wearing in public places/transportation other than workplace |  |
| Every time | 1675 (81.6) |
| Often | 280 (13.6) |
| Sometimes | 82 (4.0) |
| Never | 16 (0.8) |
| Frequency of face mask wearing when you have close contact with other people in workplace |  |
| Every time | 1519 (74.0) |
| Often | 370 (18.0) |
| Sometimes | 144 (7.0) |
| Never | 20 (1.0) |
| Self-reported sanitizing hands (using soaps, liquid soaps or alcohol-based sanitizer) after returning from public spaces or touching public installation |  |
| Every time | 1217 (59.3) |
| Often | 495 (24.1) |
| Sometimes | 323 (15.7) |
| Never | 18 (0.9) |
| Self-reported avoiding social/meal gathering with other people who do not live together |  |
| No | 1165 (56.7) |
| Yes | 888 (43.3) |
| Self-reported avoiding crowed places |  |
| No | 1309 (63.8) |
| Yes | 744 (36.2) |
|  |  |
| **COVID-19 preventive measures implemented by workplace (%Yes)** |  |
| Prohibiting non-employees entering workplaces | 1307 (63.7) |
| Taking body temperature and sanitizing hands for all employees entering the workplace | 1660 (80.9) |
| Providing facemasks to all employees | 1679 (81.8) |
| Keeping adequate distance (e.g., >1m) between work stations | 1438 (70.0) |
| Requiring employees to wear facemasks when they have close contact with other people | 1670 (81.3) |
| Frequent workplace disinfection | 1706 (83.1) |
| Maintaining adequate ventilation in workplace | 1774 (86.4) |
| Setting up partitions in factory canteens | 1335 (65.0) |
