# Supplementary material for: Behavioral Intention to Receive a COVID-19 Vaccination Among Chinese Factory Workers: Cross-sectional Online Survey
Source: J Med Internet Res. 2021 Mar 9;23(3):e24673. doi: 10.2196/24673 (PMC7945977; doi:10.2196/24673)
Supplement: Multimedia Appendix 3 [file jmir_v23i3e24673_app3.docx]

Multimedia Appendix 3

Table 1 Associations between background characteristics and behavioral intention to receive COVID-19 vaccination under different scenarios (n=2053)

|  | Conditional on efficacy in preventing COVID-19 was 80% and market rate | Conditional on efficacy in preventing COVID-19 was 80% and free vaccines |
| --- | --- | --- |
|  | OR (95%CI) | OR (95%CI) |
| Age group (years) |  |  |
| 18-30 | 1.0 | 1.0 |
| 31-40 | 0.89 (0.74, 1.07) | 1.14 (0.92, 1.41) |
| 41-50 | 0.77 (0.62, 0.94)^c^ | 0.75 (0.60, 0.94)^c^ |
| >50 | 0.55 (0.36, 0.85)^b^ | 0.86 (0.53, 1.39) |
| Gender |  |  |
| Male | 1.0 | 1.0 |
| Female | 0.96 (0.82, 1.13) | 0.88 (0.73, 1.05) |
| Relationships status |  |  |
| Currently single | 1.0 | 1.0 |
| Having a stable boyfriend/girlfriend | 1.39 (0.99, 1.95) | 1.31 (0.88, 1.94) |
| Married | 0.94 (0.78, 1.14) | 0.97 (0.78, 1.20) |
| Having children |  |  |
| No | 1.0 | 1.0 |
| Yes | 0.82 (0.69, 0.98)^c^ | 0.83 (0.68, 0.99)^c^ |
| Highest education level attained |  |  |
| Junior high or below | 1.0 | 1.0 |
| Senior high or equivalent | 1.59 (1.31, 1.91)^a^ | 2.03 (1.64, 2.51)^a^ |
| College/university or above | 1.80 (1.49, 2.19)^a^ | 2.84 (2.26, 3.58)^a^ |
| Monthly personal income (RMB) |  |  |
| <3,000 | 1.0 | 1.0 |
| 3,000-4,999 | 1.04 (0.86, 1.27) | 1.13 (0.91, 1.40) |
| 5,000-6,999 | 1.41 (1.11, 1.81)^b^ | 1.60 (1.21, 2.11)^b^ |
| 7,000-9,999 | 1.49 (1.06, 2.07)^c^ | 2.00 (1.34, 2.98)^b^ |
| ≥10,000 | 1.52 (1.06, 2.18)^c^ | 2.82 (1.75, 4.53)^a^ |
| Type of work |  |  |
| Frontline workers | 1.0 | 1.0 |
| Management staff | 1.19 (1.00, 1.42)^c^ | 1.57 (1.28, 1.93)^a^ |
| Factory type |  |  |
| Electronic devices manufacturer | 1.0 | 1.0 |
| Other factories | 1.19 (0.99, 1.48) | 1.20 (0.99, 1.47) |
| History of seasonal influenza vaccination |  |  |
| No | 1.0 | 1.0 |
| Yes | 1.48 (1.17, 1.86)^b^ | 1.17 (0.96, 1.42) |
| Having a family member with history of COVID-19 |  |  |
| No | 1.0 | 1.0 |
| Yes | 6.13 (3.27, 11.50)^a^ | 6.92 (3.70, 12.93)^a^ |

OR: crude odds ratios obtained by univariate ordinal logistic regression models

CI: confidence interval

^a^ p<0.001, ^b^ p<0.01, ^c^ p<0.05

Table 2 Factors associated with behavioral intention to receive COVID-19 vaccination under different scenarios (n=2053)

|  | Conditional on efficacy in preventing COVID-19 was 80% and market rate | Conditional on efficacy in preventing COVID-19 was 80% and free vaccines |
| --- | --- | --- |
|  | AOR (95%CI) | AOR (95%CI) |
| **Perceptions relate to COVID-19 vaccination based on the Theory of Planned Behavior** |  |  |
| Positive Attitude Scale | 1.16 (1.12, 1.21)^a^ | 1.20 (1.15, 1.24)^a^ |
| Negative Attitude Scale | 0.98 (0.93, 1.03) | 1.00 (0.96, 1.05) |
| Perceived Subjective Norm Scale | 1.49 (1.39, 1.60)^a^ | 1.42 (1.32, 1.53)^a^ |
| Perceived behavioral control to receive COVID-19 vaccination | 1.56 (1.39, 1.75)^a^ | 1.37 (1.21, 1.56)^a^ |
|  |  |  |
| **Influence of social media related to COVID-19 vaccination** |  |  |
| Frequency of exposure to positive information related to COVID-19 vaccination on social media | 1.52 (1.40, 1.66)^a^ | 1.43 (1.31, 1.57)^a^ |
| Frequency of exposure to negative information related to COVID-19 vaccination on social media | 1.09 (0.99, 1.18) | 0.99 (0.80, 1.08) |
| Frequency of exposure to testimonials given by participants of the COVID-19 vaccine clinical trials on social media | 1.09 (0.99, 1.19) | 0.93 (0.86, 1.03) |
| Frequency of exposure to negative information about vaccine incidents in China on social media | 0.95 (0.87, 1.03) | 0.91 (0.81, 1.02) |
|  |  |  |
| **Personal COVID-19 preventive measures in the past month** |  |  |
| Consistent facemask in public places/transportation other than workplace |  |  |
| No | 1.0 | 1.0 |
| Yes | 1.65 (1.35, 2.03)^a^ | 1.26 (1.00, 1.70)^c^ |
| Consistent facemask when you have close contact with other people in workplace |  |  |
| No | 1.0 | 1.0 |
| Yes | 1.46 (1.21, 1.75)^a^ | 1.34 (1.08, 1.67)^b^ |
| Self-reported sanitizing hands (using soaps, liquid soaps or alcohol-based sanitizer) every time after returning from public spaces or touching public installation |  |  |
| No | 1.0 | 1.0 |
| Yes | 1.45 (1.23, 1.70)^a^ | 1.36 (1.12, 1.63)^b^ |
| Self-reported avoiding social/meal gathering with other people who do not live together |  |  |
| No | 1.0 | 1.0 |
| Yes | 1.17 (1.00, 1.37)^c^ | 1.49 (1.24, 1.78)^a^ |
| Self-reported avoiding crowed places |  |  |
| No | 1.0 | 1.0 |
| Yes | 1.21 (1.02, 1.42)^c^ | 1.65 (1.37, 1.99)^a^ |
|  |  |  |
| **COVID-19 preventive measures implemented by workplace** |  |  |
| Number of preventive measures implemented by the factory | 1.09 (1.06, 1.13)^a^ | 1.07 (1.03, 1.10)^a^ |

AOR: adjusted odds ratios, background characteristics with p<0.05 in univariate ordinal logistic regression analysis were adjusted in the multivariate ordinal logistic regression models

CI: confidence interval

^a^ p<.001, ^b^ p<.01, ^c^ p<.05
