# Supplementary material for: Behavioral Intention to Receive a COVID-19 Vaccination Among Chinese Factory Workers: Cross-sectional Online Survey
Source: J Med Internet Res. 2021 Mar 9;23(3):e24673. doi: 10.2196/24673 (PMC7945977; doi:10.2196/24673)
Supplement: Multimedia Appendix 4 [file jmir_v23i3e24673_app4.docx]

Multimedia Appendix 4

Table 1 Correlations between information exposure through social media exposure and perceptions related to COVID-19 vaccination based on the Theory of Planned Behavior

| Social media exposure | Positive Attitude Scale | Negative Attitude Scale | Perceived Subjective Norm Scale | Perceived behavioral control to take up COVID-19 vaccination |
| --- | --- | --- | --- | --- |
|  | r ^a^ (p values) | r ^a^ (p values) | r ^a^ (p values) | r ^a^ (p values) |
| Frequency of exposure to positive information related to COVID-19 vaccination on social media | 0.083  (p<.001) | -0.090  (p <.001) | 0.101  (p <.001) | 0.064  (p=0.004) |
| Frequency of exposure to negative information related to COVID-19 vaccination on social media | -0.080  (p<.001) | 0.013  (p=.55) | -0.107  (p<.001) | -0.069  (p=.002) |
| Frequency of exposure to testimonials given by participants of the COVID-19 vaccine clinical trials on social media | -0.052  (p=.02) | -0.023  (p=.30) | -0.037  (p=.09) | 0.062  (p=.005) |
| Frequency of exposure to negative information about vaccine incidents in China on social media | -0.106  (p<.001) | 0.029  (p=.19) | -0.132  (p<.001) | -0.030  (p=.17) |

^a^ r: Pearson correlation coefficient
